# Plants use molecular mechanisms mediated by biomolecular condensates to integrate environmental cues with development

**DOI:** 10.1093/plcell/koad062

**Published:** 2023-03-05

**Authors:** Sterling Field, Geng-Jen Jang, Caroline Dean, Lucia C Strader, Seung Y Rhee

**Affiliations:** Department of Plant Biology, Carnegie Institution for Science, Stanford, CA 94305, USA; Department of Cell and Developmental Biology, John Innes Centre, Norwich Research Park, Norwich NR4 7UH, UK; Department of Cell and Developmental Biology, John Innes Centre, Norwich Research Park, Norwich NR4 7UH, UK; Department of Biology, Duke University, Durham, NC 27708, USA; Department of Plant Biology, Carnegie Institution for Science, Stanford, CA 94305, USA

## Abstract

This review highlights recent literature on biomolecular condensates in plant development and discusses challenges for fully dissecting their functional roles. Plant developmental biology has been inundated with descriptive examples of biomolecular condensate formation, but it is only recently that mechanistic understanding has been forthcoming. Here, we discuss recent examples of potential roles biomolecular condensates play at different stages of the plant life cycle. We group these examples based on putative molecular functions, including sequestering interacting components, enhancing dwell time, and interacting with cytoplasmic biophysical properties in response to environmental change. We explore how these mechanisms could modulate plant development in response to environmental inputs and discuss challenges and opportunities for further research into deciphering molecular mechanisms to better understand the diverse roles that biomolecular condensates exert on life.

## Introduction

Plants continuously generate new organs throughout their life cycle while constantly interpreting and acclimating to changing environments. This acclimation includes both physiological responses and developmental changes that allow plants to interpret changing environments over temporal scales ranging from seconds to years, enabling optimal survival and reproduction. Research in plant development initially focused on master regulators that cause obvious phenotypes when a single gene is mutated (Koornneef and Meinke 2010). Building on this, our more recent understanding of development has centered on how these regulators function: gene activation or repression, translational control, transcript degradation, protein modifications, and regulation of multimeric protein complexes. However, how multivalent interactions can act as major contributors to plant development has remained controversial (Lorković 2009; Merchante et al. 2017; Song et al. 2019). Answers to this issue have emerged through the realization that protein complexes form subcellular membraneless compartments termed biomolecular condensates. Biomolecular condensates are an umbrella term used to group membraneless cellular compartments formed by diverse proteins and ligands that function to compartmentalize reactions and signaling pathways in organisms (Banani et al. 2017; Lyon et al. 2020; Emenecker et al. 2021). Biomolecular condensates can form and dissolve via modulated multivalent interactions of biomolecules, assisting in rapid cellular changes in response to the environment.

Biomolecular condensate formation is involved in numerous processes, including transcription, RNA processing, translation, cellular signaling, and metabolism. Frequently, the role of biomolecular condensates is dominated by the discussion of well-characterized examples like mRNA processing and mRNA storage in processing bodies or stress granules (Protter and Parker 2016; Luo et al. 2018; Hofmann et al. 2021). While these examples provide excellent insight into regulatory mechanisms of biomolecular condensates, focusing exclusively on them both obfuscates the general mechanisms that biomolecular condensates perform in the cell and over-shadows the diversity of proteins and pathways in which biomolecular condensates are involved.

**Figure 1.**
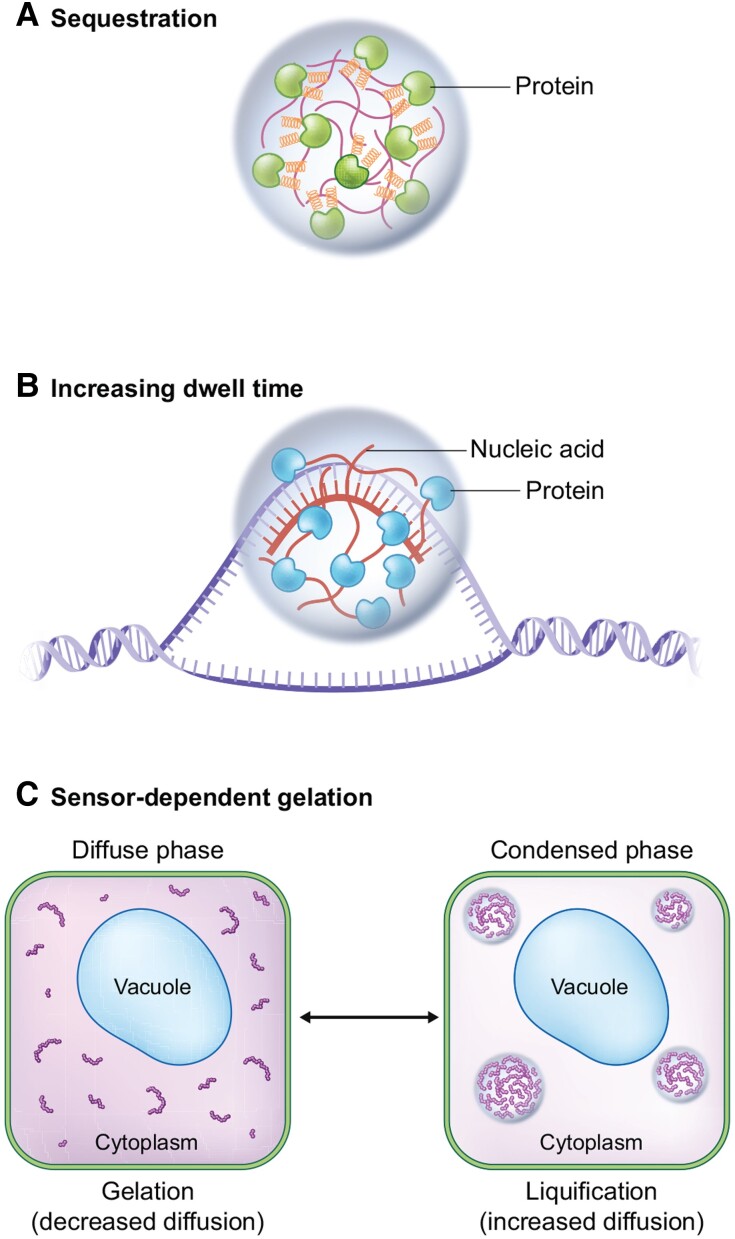
General molecular mechanisms of biomolecular condensates. **A)** Sequestration of pathways away from ligands or interacting partners. Biomolecular condensates “hold” proteins or nucleic acids away from their targets or interacting partners not in the condensate. An example of this mechanism is mediated by the processing body and its components (green proteins) that help sequester translationally repressed mRNAs (red line in condensate). During skotomorphogenesis, this phenomenon may be required for the protein DCP5. When these nontranslating mRNAs in processing bodies are released, these mRNAs may undergo translation. **B)** Increasing dwell time of reaction components. Biomolecular condensates increase the local concentration of proteins (blue RNA-binding proteins) or nucleic acids (red nucleic acid) at a site to enhance a reaction or signaling pathway. An example of this regulation is mediated by FCA condensates that compartmentalize 3′ processing factors and help resolve the R-loop formed by the *FLC* antisense transcript (*COOLAIR*) at the *FLC* locus. **C)** Condensation mediated changes to cellular biophysical properties. Biomolecular condensate formation or dissolution can change biophysical properties of the cytoplasm, including changes to viscosity and protein diffusion. No published examples of this mechanism are available in plants at this time. The figure shows biomolecular condensate formation resulting in cytoplasmic liquification, based on the current hypothesis for FLOE1 function. The opposite is observed with Tardigrade CAHS, where CAHS condensate formation results in cytoplasmic gelation.

This review summarizes the current literature on biomolecular condensates involved in plant development, how biomolecular condensates may exert their functions in shaping development, and challenges for future research investigating their molecular mechanisms. The roles of individual biomolecular condensates in development are diverse. We group recent examples into 3 categories of mechanisms (Fig. 1). First, biomolecular condensates sequester proteins (or ligands, e.g. RNA) away from their target, preventing a reaction or signal from occurring (Banani et al. 2017; Lyon et al. 2020; Roden and Gladfelter 2020). This mechanism allows biomolecular condensates to inhibit reactions through their formation, or to enhance interactions when the condensate is dissolved and sequestered components are released. Second, biomolecular condensate formation increases dwell time of components to enhance interactions and avidity (see definitions of useful terms in Table 1). Increased dwell time is the result of high, local concentrations of biomolecules in a condensed state, reducing the probability of binding partners from diffusing away (reviewed in Banani et al. 2017; Bienz 2020; Lyon et al. 2020; Roden and Gladfelter 2020). Dwell times are primarily determined by efficient rebinding of partners rather than diffusion. This mechanism can enhance the rate, equilibrium, duration, or specificity of interactions within the condensate, and serve as an additional layer of pathway regulation (additional mechanisms and their examples discussed in Case et al. 2019). Third, biomolecular condensates may interact with cytoplasmic biophysical properties of the cell, such as cytoplasmic stiffness, during stress (Tanaka et al. 2022).

**Table 1.** Biomolecular condensate concepts and terminology

Concepts and terms	Definitions
Biomolecular condensates	Biomolecular condensates are nonmembrane bound concentrations of biomolecules that can exist in varying states, including liquid-, gel-, or solid-like states that differ from what would normally be a diffuse state, under physiological or in vivo conditions.
Phase separation	Phase separation is a term describing a mechanism by which a biomolecular condensate may form, driven by physical principles of molecules transitioning from a 1-phase to a 2-phase regime (see Emenecker et al. 2021). Phase separation can occur in vivo, in vitro, or in nonphysiological conditions.
Avidity vs affinity	Both are measures of binding strength. Affinity is the binding strength of a single bond or interaction, while avidity is the strength of all binding/interacting sites. Avidity is commonly used in this field because of the requirement for multivalent biomolecular interactions driving condensate formation. Avidity is more appropriate for describing ligands interacting with multiple binding partners in a condensate, compared to the affinity of a single protein–ligand interaction.
**Protein domains involved in biomolecular condensate formation**	
IDR	A domain or region of the protein that does not adopt a stable ordered secondary structure.
Prion-like domain	A low complexity IDR protein domain that lacks the secondary structure that is similar to published prion domains.
PB1 domain	A ubiquitin beta-grasp fold domain that drives protein–protein assembly of ARF19.
MR domain	An IDR in ARF19 required for ARF19 condensate formation.
Hinge region	A disordered RNA binding domain required for LHP1 condensate formation.
DIX-like domain	Similar to domains identified in oligomerization of the DISHEVELLED and AXIN proteins, composed of an alpha-helical and a beta-sheet structure.
SMP domain	An alpha-helical domain that drives LEA condensation.
QPS domain	A prion-like IDR required for FLOE1 hydration-dependent condensate formation.
DS domain	An IDR required for preventing FLOE1 protein aggregates.

These mechanisms can help explain how evolution has selected for multivalent interactions to work together to progressively pass biochemical checkpoints through biomolecular condensates in many developmental processes. These mechanisms also provide a rationale for how organisms might extract signals from noisy environmental conditions. Further, they provide insight into how cells maintain concentrations of factors at specific sites despite fluctuations in expression or degradation, thus decreasing “noise” from the biological system (Banani et al. 2017).

**Figure 2.**
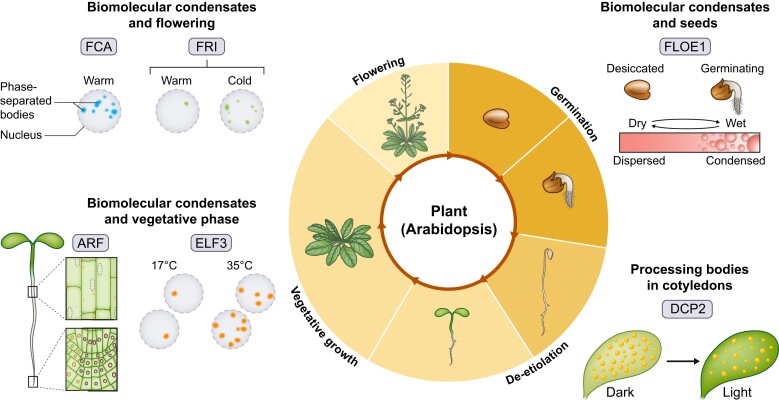
Summary of biomolecular condensates in plant development. Clockwise from upper right; GERMINATION: FLOE1 is essential for coordinating germination to appropriate environmental conditions. FLOE1 forms condensates upon addition of water to regulate germination in seed cells. DE-ETIOLATION: processing bodies in cotyledons decrease in number in response to light and help young seedlings timely modify morphogenesis. VEGETATIVE GROWTH: ARF19 undergoes nucleo-cytoplasmic shuttling, and biomolecular condensate formation dampens auxin response, regulating root growth. ELF3 forms nuclear localized condensates in hypocotyl cells in response to high temperatures. FLOWERING: multiple biomolecular condensates including FCA and FRIGIDA (FRI) regulate expression of a key repressor to fine-tune the timing of flowering. FCA forms nuclear condensates that are not temperature-responsive, while FRI nuclear condensates become larger and more stable in response to cold.

Recent work has identified biomolecular condensates in plant development, with roles spanning the life cycle of the plant, ranging from germination through vegetative growth and flowering (Fig. 2). We discuss studies of biomolecular condensates at different stages of plant development, grouped by types of mechanisms, based on the current state of research. We also highlight protein domains involved in biomolecular condensate formation and discuss how manipulating these regions could link condensate formation with function.

## Biomolecular condensates involved in vegetative development

Analyses of many aspects of vegetative development involving photoreceptor biology, hormone signaling, and circadian clock function have identified examples where biomolecular condensates function through sequestration and increasing dwell time.

### Sequestration of nontranslating mRNAs in processing bodies during skotomorphogenesis

The function of processing bodies during skotomorphogenesis is to sequester selected mRNAs and prevent their translation. Skotomorphogenic development occurs after seeds germinate underground. Seedlings growing in darkness have elongated hypocotyl, closed cotyledons, and an apical hook. When seedlings protrude from soil and sense light, plants undergo morphological changes termed photomorphogenesis, a process involving cotyledon greening, apical hook opening, and inhibition of hypocotyl growth.

Processing bodies are cytoplasmic condensates that function in RNA decay and translational repression in many organisms (Luo et al. 2018; Jang et al. 2020). Loss of function of DECAPPING PROTEIN 5 (DCP5), an RNA-binding protein and one of the Arabidopsis (*A. thaliana*) processing body components, decreases the number of DCP1 and DCP2 marked processing bodies (Xu and Chua 2009; Jang et al. 2019). *dcp5* mutants grown in the dark have increased translation efficiency of hundreds of mRNAs, impacting skotomorphogenesis development and apical hook maintenance. After seedlings reach the soil surface, DCP2-marked processing bodies decrease in size and number in cotyledons through a phytochrome-dependent manner. The processing body-associated and translationally repressed mRNAs are then released for synthesizing proteins, including chlorophyll biosynthetic enzymes and putative auxin carriers essential for photomorphogenesis and adaptation to light (Jang et al. 2019).

Recent studies highlight the tight association between phytochromes and processing bodies during photomorphogenesis. Phytochromes are red- and far-red light receptors that control many biological processes including germination, photomorphogenesis, shade avoidance, flowering, and dormancy (Galvão and Fankhauser 2015). The phytochromobilin synthase encoded by *LONG HYPOCOTYL 2* (*HY2*) is essential for synthesizing the phytochrome chromophore phytochromobilin and making functional phytochrome (Kohchi et al. 2001). Light-triggered reduction of processing bodies does not occur in the light-insensitive *hy2-106* mutant (Jang et al. 2019). The CONSTITUTIVELY PHOTOMORPHOGENIC 1/SUPPRESSOR OF PHYA-105 (COP1/SPA) complex is a central repressor in the phytochrome-mediated signaling pathways (Saijo et al. 2003). Loss of function of COP1 results in a photomorphogenic phenotype and increased translation in dark-grown seedlings (Lau and Deng 2012; Chen et al. 2018). Consistent with the phenotypes and the increased translation status of the *cop1* mutant, processing bodies do not accumulate in etiolated *cop1* seedlings (Jang et al. 2019).

One additional connection between phytochromes and processing body regulation is NOT9B. NOT9B is a phytochrome A (phyA) interacting protein identified as a negative regulator of light signaling and is a component of CARBON CATABOLITE REPRESSION 4-NEGATIVE ON TATA-LESS (CCR4-NOT) complex (Schwenk et al. 2021). NOT9B colocalizes with DCP1 marked processing bodies in tobacco and Arabidopsis seedlings (Schwenk et al. 2021). NOT9B biomolecular condensate number is reduced upon exposure to far-red and red light and coincides with the reduction of processing body markers in response to far-red light. Further, cytoplasmic phyA is essential for mediating far-red light-dependent reduction of processing bodies and hypocotyl growth (Schwenk et al. 2021; Schwenk and Hiltbrunner 2022). Based on current findings, processing bodies accumulate in dark-grown seedlings and function in sequestering nontranslated mRNAs. The reduction of processing bodies is regulated by light signaling pathways to ensure that selected mRNAs are translated at a proper time during the dark-light transition.

### EARLY FLOWERING 3, a component of the circadian clock, is sequestered into biomolecular condensates in response to light and temperature

The circadian clock is an internal biological system to regulate plant physiology through integrating temperature and light signals (Millar 2004). The EARLY FLOWERING 3 (ELF3) protein contains a prion-like domain and forms biomolecular condensates in response to changes in temperature and light (Nusinow et al. 2011; Jung et al. 2020; Ronald et al. 2022). Warm temperatures enhance ELF3 condensate formation in hypocotyl cells in the afternoon but not in the morning (Murcia et al. 2022). ELF3 acts as a scaffold protein and the scaffold activity assists in recruiting components such as ELF4 and LUX ARRHYTHMO (LUX) to the circadian clock evening complex (Nusinow et al. 2011; Herrero et al. 2012; Jung et al. 2020). ELF3 colocalizes with phyB and TANDEM ZINC-FINGER/PLUS3 (TZP), an integrator of light and photoperiodic pathways, in nuclear photobodies (Kaiserli et al. 2015). ELF3 condensates accumulate in the dark, and the accumulation is further induced by prior blue light treatment while reduced with prior red-light treatment (Ronald et al. 2022). Functionally, ELF3 condensates repress ELF3 activity during high temperatures through a decrease in ELF3 binding to its target genes (Jung et al. 2020), suggesting ELF3 condensates serve to sequester ELF3 activity.

### Biomolecular condensates are essential to sequester hormone signaling transcription factors

Hormone signaling is an essential patterning mechanism for plant development. Spatial control of auxin transcriptional output is critical for driving plant developmental events, such as leaf initiation, apical hook formation, lateral root production, and root apical meristem maintenance. Recent work identified biomolecular condensate formation of proteins in the auxin hormone signaling pathways (Powers et al. 2019; Jing et al. 2022), tying together spatial regulation of the auxin response in roots with biomolecular condensate formation.

AUXIN RESPONSE FACTOR 19 (ARF19) is a transcription factor that forms condensates essential for regulating its own activity. ARF19 undergoes nucleo-cytoplasmic cycling; in the nucleus ARF19 is diffuse while in the cytoplasm ARF19 forms condensates (Powers et al. 2019). ARF19 condensate formation in roots changes along the length of the root and is critical for regulating root development. In tissues with ARF19 cytoplasmic condensates, there is a dampened auxin transcriptional response compared to other tissues without ARF19 condensates, suggesting ARF19 is sequestered to prevent auxin signaling.

ARF19 condensate formation is driven by protein–protein interactions of the ubiquitin beta-grasp fold Phox and Bem1 (PB1) domain and requires the intrinsically disordered region (IDR) called middle region (MR) domain (Powers et al. 2019). Mutants of ARF19 that do not form condensates, ARF19^K962A^, have transcript populations upregulated similarly to WT with auxin treatment (Powers et al. 2019), supporting the hypothesis that the ARF19 condensates inhibit ARF19 function via sequestration. ARF19^K962A^ mutants display altered root development indicative of hyperactive auxin responsiveness. ARF19 condensates are further regulated by degradation through the Ub-proteasome and require the E3 ubiquitin ligase SCF^AFF1^ (Jing et al. 2022). Together, formation of ARF19 condensates function to sequester the ARF19 transcription factor away from the nucleus to prevent or dampen auxin signaling in root development.

### Increased local concentration of photoreceptors and circadian clock components in biomolecular condensates

Phytochrome activity is regulated by its subcellular localization in which the protein forms nuclear speckles after light exposure (Mackenzie et al. 1975). Photo-activated phytochrome B (phyB) rapidly translocates from the cytoplasm to the nucleus and forms condensates (called photobodies) dependent on light wavelength and intensity (Sakamoto and Nagatani 1996; Kircher et al. 1999; Chen et al. 2003). PhyB integrates light and temperature signals through changes to liquid–liquid phase separation (LLPS) (Chen et al. 2022; see other articles in this issue for additional information on photobodies).

Cryptochromes are another group of photoreceptors that have numerous functions, including perceiving blue light and mediating the photomorphogenesis and circadian clock in plants (Wang and Lin 2020). Cryptochromes regulate the circadian clock through the CRYPTOCHROME 2 (CRY2) protein localizing to biomolecular condensates. CRY2 forms liquid–liquid phase separated biomolecular condensates only after first being activated by blue light (Más et al. 2000). Functionally, cryptochromes regulate m^6^A RNA modification on more than 10% of mRNAs in Arabidopsis young seedlings, and the photo-activated CRY2 condenses m^6^A writer proteins by multivalent interactions (Wang et al. 2021a), suggesting CRY2 condensates function to modify mRNA. CRY2 regulates the circadian clock through CIRCADIAN CLOCK ASSOCIATED 1 (CCA1), a component of the central oscillator that forms transcriptional feedback loops (Wang and Tobin 1998). The TEOSINTE BRANCHED1-CYCLOIDEA-PCF 22 (TCP22) transcription factor and LIGHT-REGULATED WD1 (LWD1) form a complex to activate *CCA1* expression by associating at the *CCA1* promoter (Wu et al. 2016). TCP22 proteins localize to CRY2 condensates in plant cells after blue light exposure (Mo et al. 2022). LWD1 prefers to interact with phosphorylated TCP22. In line with this, CRY2-TCP22 condensates are maintained by PHOTOREGULATORY PROTEIN KINASE 1 mediated phosphorylation at the *CCA1* promoter region. Together, these 4 proteins colocalize into condensates in protoplasts (Mo et al. 2022), which may increase dwell time to activate *CCA1* expression for regulating the circadian clock, though direct evidence is currently lacking.

### Other developmental programs also involve RNA processing and biomolecular condensation

Several stress-associated RNA processing pathways localize to biomolecular condensates and have possible roles in development. For instance, several miRNAs target developmental patterning genes, and dicing bodies and the protein SERRATE are critical for miRNA biogenesis (Grigg et al. 2005; Lobbes et al. 2006). Similarly, trans-acting siRNAs (tasiRNAs) regulate several ARFs involved in development. tasiRNA biogenesis requires SUPPRESSOR OF GENE SILENCING 3, which is essential for forming siRNA bodies (Kim et al. 2021). siRNA bodies and dicing bodies both increase concentration and dwell time of their components for small RNA biogenesis.

SERRATE was previously established to regulate miRNA processing, including miR165 and miR166 that repress the abaxial/adaxial patterning genes PHABULOSA and PHAVOLUTA (Grigg et al. 2005; Lobbes et al. 2006). Dicing bodies are miRNA processing centers and require phase separation of SERRATE for assembly (Xie et al. 2021). SERRATE is a zinc-finger protein essential for lateral organ development, shoot elongation, and leaf patterning (Prigge and Wagner 2001). In dicing bodies, SERRATE processes primary and precursor miRNAs into mature miRNAs that are then released to silence targets (Xie et al. 2021). Overall, miRNAs have been established to impact development and miRNA processing is regulated by biomolecular condensates. However, experimental evidence directly connecting biomolecular condensates with miRNA-mediated development has not yet been demonstrated.

## Biomolecular condensates involved in flowering

The regulation of flowering has identified numerous biomolecular condensates, including some that appear to exert antagonistic mechanisms of sequestering components and increasing dwell time on a single pathway.

### Regulation of flowering time by TMF

TERMINATING FLOWER (TMF) is a transcription factor that prevents premature maturation of the shoot apical meristem into floral transition. TMF will bind and form condensates at the promoter of the *ANANTHA* F-Box floral identity gene, sequestering the promoter from transcriptional activators and preventing expression (Huang et al. 2021). TMF biomolecular condensation is driven by local accumulation of reactive oxygen species (ROS; e.g. H_2_O_2_) that forms disulfide bridges between TMF proteins and condenses TMF. Four cysteine residues in TMF's IDR are essential for modulating this phase separation through their oxidation (Huang et al. 2021). This ROS-mediated regulation of TMF alters *ANATHA* expression to regulate early floral meristem formation (Allen and Sussex 1996; Lippman et al. 2008).

### Regulation of *FLOWERING LOCUS C* by sequestering regulators

FLOWERING LOCUS C (FLC) is a key flowering repressor in Arabidopsis. FLC is a MADS-Box transcription factor that regulates hundreds of genes (Deng et al. 2011), including *FLOWERING LOCUS T* and *SUPPRESSOR OF OVEREXPRESSION OF CO 1,* which control the timing of flowering (Helliwell et al. 2006; Searle et al. 2006). Therefore, regulation of *FLC* expression is an important mechanism for plants to transition from a vegetative to reproductive phase of development at the appropriate time. Recent work indicates that *FLC* is both positively and negatively regulated via biomolecular condensates.

FRIGIDA (FRI) is an important positive regulator of *FLC* expression, which ensures plants overwinter before flowering. FRI associates with FRIGIDA-LIKE proteins, transcription factors, and splicing factors that all promote *FLC* expression cotranscriptionally. FRI nuclear condensates form in response to cold exposure (∼6 h after plants are moved to cold). FRI condensates are relatively stable, and dissolve in a similar timeframe upon return to warm temperatures, features partly associated with changed FRI stability and association with a cold-specific isoform of the *FLC* antisense transcript (Zhu et al. 2021). The FRI disordered and coiled-coil domains are required for condensate formation, which are enriched with FRIGIDA-LIKE1, EARLY FLOWERING 7, and the Cajal body marker U2 SMALL NUCLEAR RIBONUCLEOPROTEIN (Zhu et al. 2021). FRI condensates do not associate with the transcriptionally active *FLC* locus (Zhu et al. 2021) and may sequester cotranscriptional activators away from the *FLC* locus. The cold-induced increase in FRI condensates correlates with the decreased expression of *FLC* (Zhu et al. 2021). Therefore, the condensation mechanism has the potential to dynamically partition the FRI cotranscriptional activators, conferring plasticity to *FLC* regulation in response to natural temperature fluctuations. However, direct evidence for this mechanism is currently lacking.

Biomolecular condensate formation is also involved in the epigenetic silencing of *FLC* expression by prolonged cold, a process called vernalization. VERNALIZATION 1 (VRN1) is a nonsequence-specific DNA-binding protein required for vernalization (Levy et al. 2002; Sung and Amasino 2004). VRN1 forms biomolecular condensates in vitro and requires DNA for condensate formation (Wang et al. 2021b). This may influence VRN1 association with DNA in vivo since VRN1 associates with DNA over all 5 mitotic Arabidopsis chromosomes (Mylne et al. 2006). LIKE HETEROCHROMATIN PROTEIN 1 (LHP1) is also involved in *FLC* silencing. LHP1 forms dynamic nuclear condensates, somewhat similarly to its mammalian homologue HP1 (Berry et al. 2017). An RNA-binding hinge region of LHP1 is required for this condensate formation and *FLC* repression (Berry et al. 2017). In Arabidopsis, LHP1 associates with Polycomb-Repressive Complex 2 (PRC2) and is required for spreading the classic histone modification delivered by the PRC2 complex, trimethylation of lysine 27 on the histone 3 tail (H3K27me3), across the *FLC* locus. This maintains the silenced state of *FLC* as the plant returns to warm conditions (Mylne et al. 2006; Sung et al. 2006; Derkacheva et al. 2013; Yang et al. 2017). Whether LHP1 condensation sequesters the silenced loci in the Arabidopsis nucleus into what has been termed Polycomb bodies in mammalian cells is still to be established.

### Increased dwell time mechanisms potentially influencing flowering through *FLC* regulation

FLOWERING CONTROL LOCUS A (FCA) represses *FLC* expression to promote a rapid-cycling strategy in Arabidopsis. FCA is an RNA-binding protein containing a prion-like domain and is localized in liquid-like nuclear condensates (Fang et al. 2019), together with many of the conserved 3′ RNA processing and polyadenylation factors (Fang et al. 2019). FCA promotes 3′ processing of an *FLC* antisense transcript (called *COOLAIR*) at a proximal site (Liu et al. 2010). This proximal termination of transcription resolves an R-loop, a 3 stranded DNA–RNA hybrid nucleic acid structure, formed by antisense transcription. This clears a potential “tangle” in the chromatin before the next transcription event or DNA replication occurs and delivers a chromatin environment that reduces transcription (Liu et al. 2010; Sun et al. 2013; Fang et al. 2020; Xu et al. 2021b). FCA/3′ processing condensates appear to increase dwell time of the polyadenylation factors at the R-loop region. The in vivo significance of this mechanism was revealed by mutants influencing FCA/3′ processing condensate dynamics. These mutants identified a role for several proteins, including the coiled-coil protein FLL2, enzymes for N^6^-methyladenosine (m^6^A) RNA modification of *COOLAIR* and with m^6^A methyltransferase function, and ARGONAUTE1 (AGO1), a protein historically considered to function in small RNA regulation (Fang et al. 2019; Xu et al. 2021a). FCA activity at *FLC* functions to antagonize the promotion of *FLC* expression by FRI (discussed above). Interestingly, while temperature regulates FRI condensate formation, the liquid-like FCA condensation is surprisingly unaffected by cold (Zhu et al. 2021). In summary, 2 genetically antagonistic regulators of *FLC* form biomolecular condensates with different biophysical properties and are controlled by different inputs. These condensates are associated with different phases of the transcription cycle and would not necessarily be operating simultaneously. The functional coordination of biomolecular condensates that both activate and repress the same target is an interesting future area to explore.

Another regulator of *FLC* is the RNA-binding protein hnRNP R-LIKE PROTEIN (HRLP). HRLP forms phase-separated condensates with the splicing factor ARGININE/SERINE-RICH 45 (SR45) and promotes R-loop formation of sense *FLC* (Zhang et al. 2022). This increased R-loop formation reduces RNA polymerase II recruitment, thus lowering *FLC* expression (Zhang et al. 2022). HRLP with truncated IDRs did not form condensates and had reduced R-loop formation, along with an increase in *FLC* expression (Zhang et al. 2022). This suggests that HRLP condensates may increase the dwell time of HRLP and SR45 to enhance R-loop formation, which suppresses *FLC* expression.

A distinct protein polymerization mechanism involving head–tail polymer interactions also targets *FLC* to epigenetically silence it during vernalization. Two Arabidopsis PRC2 accessory proteins, VERNALIZATION INSENSITIVE 3 and VERNALIZATION 5 (VEL proteins), contain a protein interaction domain unrelated to the well-characterized SAM and PB1 domains that are capable of spontaneous head-to-tail polymerization (Greb et al. 2007; Fiedler et al. 2022). Mutations with just 1 amino acid change in the VEL polymerization interface prevent Polycomb silencing at *FLC* (Fiedler et al. 2022). Plant VEL proteins likely increase the dwell time of PRC2 complexes at their targets by increasing the rate of rebinding after their dissociation (Bienz 2020), a mechanism well-established in many biological systems but difficult to test in planta. The high concentration of VEL proteins at the genomic nucleation sites may hold PRC2 at target loci to maintain epigenetic silencing through many cell cycles and over long developmental periods.

## Biomolecular condensates involved in embryogenesis and germination

Embryo development and seed germination involve several biomolecular condensates. One well-characterized example exerts an increased dwell time mechanism, while the mechanism of other condensates observed during germination remains controversial.

### SOSEKI condensates in cellular polarity

One of the best examples of the importance of biomolecular condensates in developmental regulation is SOSEKI (SOK), a protein involved in cell polarity in plants (van Dop et al. 2020). SOK proteins show a striking polar localization in various Arabidopsis tissues to control the orientation of cell division (Yoshida et al. 2019). SOK proteins form dynamic biomolecular condensates in Arabidopsis embryos and are associated with cell membranes specifically on one corner of the cell. These condensates form large aggregates through a DIX-like protein polymerization domain, paralleling the VEL polymerization mechanism, discussed above. SOK polymerization is required for recruitment of a putative effector protein called ANGUSTIFOLIA. ANGUSTIFOLIA is critical for arrangement of microtubules to regulate cell wall expansion in developing leaves (Kim et al. 2002).

### Condensates interact with cytoplasmic biophysical properties

Another group of biomolecular condensates has been observed during seed germination, but the molecular mechanisms they perform remain unknown. Currently, there is debate about whether changes to cytoplasmic properties drive biomolecular condensation, or if biomolecular condensation can drive changes to cellular biophysical properties. For example, changes in ribosome concentration mediated by mTORC1 resulted in changes to molecular crowding and cytoplasmic viscosity, which resulted in changes to biomolecular condensate formation (Delarue et al. 2018). An argument for biomolecular condensates driving changes to biophysical properties was made based on a protein that forms biomolecular condensates in tardigrades, which are multicellular organisms that remain viable after undergoing extreme desiccation (Tanaka et al. 2022). Oligomerization of members of the tardigrade CYTOPLASMIC ABUNDANT HEAT SOLUBLE PROTEIN (CAHS) family drives gelation of the cytoplasm during water stress (Tanaka et al. 2022). Members of CAHS are freely dispersed in the cytoplasm but low water stress results in CAHS reversibly forming filament-like condensates that drive gelation of the cytoplasm (Tanaka et al. 2022). CAHS expressed in heterologous systems resulted in cytoplasmic gelation, enhanced cell stiffness, and prevented cellular deformation during low water stress (Tanaka et al. 2022).

A major direction for future research in this area is to better connect the cause and effect of biomolecular condensate formation and how this impacts cellular biophysical properties. Explicitly connecting biomolecular condensate regulation with other biophysical properties will require further research in the broader biomolecular condensates field. There have not been any published examples of plant proteins forming biomolecular condensates to drive changes to the cellular biophysical properties, but based on current literature, we discuss 2 possible candidates: LATE EMBRYO ABUNDANCE (LEA) proteins and FLOE1.

### Connecting germination to condensate-mediated changes in cellular biophysical properties

The plant life cycle requires cells to undergo a programmed desiccation. During seed development, the developing embryo desiccates to reach the mature seed stage. Upon favorable environmental conditions, the dried, mature seed will germinate, which requires cells to rehydrate to resume metabolism (Rajjou et al. 2012; Nonogaki 2014; Carrera-Castaño et al. 2020). FLOE1 and the LEA proteins have been implicated in desiccation during embryo development and sensing water during seed germination, respectively (Hundertmark and Hincha 2008; Dorone et al. 2021).

LEA proteins contain prion-like domains, are implicated in desiccation and viability of desiccated cells, and have been identified throughout eukaryotes (Tunnacliffe and Wise 2007; Battaglia et al. 2008; Hundertmark and Hincha 2008). LEA proteins are a broad group of disordered hydrophilic proteins composed of 4 subgroups, 2 of which are found only in plants (Tunnacliffe and Wise 2007; Hesgrove and Boothby 2020). LEA proteins can prevent other proteins from aggregating during desiccation and low water stress (Chakrabortee et al. 2012; Belott et al. 2020). Ectopic expression of LEA proteins enhances low water stress survival in cells, decreases protein aggregation during heat and low water stress, and protects enzyme activity (Goyal et al. 2005; Dang et al. 2014; Belott et al. 2020). Under low water stress, LEA proteins form biomolecular condensates driven by the alpha-helical seed maturation protein (SMP) domain (Belott et al. 2020). While the molecular mechanism behind LEA proteins’ ability to prevent aggregation remains elusive, it was recently proposed to be through modulating changes in the biophysical properties of the environment around proteins (Chakrabortee et al. 2012; Belott et al. 2020).

Arabidopsis has 51 LEA proteins (Hundertmark and Hincha 2008), though the formation of condensates has only been demonstrated in a few members of the LEA subgroup 4 (Ginsawaeng et al. 2021). In subgroup 4, LEA9, LEA48, and LEA42–LEA48 heterodimers formed condensates (Ginsawaeng et al. 2021). LEA9 forms biomolecular condensates in dry seeds, while LEA9 in hydrated cells forms biomolecular condensates only in low water stress conditions. LEA9 condensates disappear within 24 h in hydrated seeds (Ginsawaeng et al. 2021). Further research is required to test if LEA9 condensates modulate changes to biophysical properties of cells during seed desiccation and rehydration.

Another candidate plant protein that could drive changes to cytoplasmic biophysical properties through biomolecular condensate formation is FLOE1. FLOE1 is expressed and forms condensates during embryo development. FLOE1 contains a prion-like domain and localizes diffusely in dry seeds but rapidly (less than a minute) forms biomolecular condensates upon addition of water (Dorone et al. 2021). Mutants lacking FLOE1 (*floe1-1*) have increased germination rate under low water stress conditions compared to the wildtype, indicating FLOE1 is essential for regulating germination in response to environmental conditions (Dorone et al. 2021).

FLOE1 biomolecular condensate formation is critical for regulating germination during low water stress. FLOE1 requires the prion-like glutamine-, proline-, serine-rich (QPS) domain for forming condensates in response to hydration and regulating seed germination (Dorone et al. 2021). Deletion of the disordered aspartic acid- and serine-rich (DS) domain of FLOE1 results in large, solid, FLOE1 protein aggregates, and higher germination rate in salt conditions than wildtype and *floe1-1* plants (Dorone et al. 2021). These observations suggest FLOE1 biomolecular condensate formation is essential for regulating germination. What remains unknown is how FLOE1 performs these functions and if FLOE1 alters cytoplasmic biophysical properties during desiccation and rehydration of seed cells.

## Challenges for the field of biomolecular condensates in plants

The biggest challenge for understanding the role and function of biomolecular condensation in plant development is to connect them with molecular, biochemical, and biophysical mechanisms. We begin this section by listing what we consider the most pressing open questions about the functions of biomolecular condensates in regulating plant growth and development, followed by sections that address technical challenges slowing progress in the field. Tools and methodologies that will help us overcome these challenges are summarized in Table 2.

**Table 2.** Techniques and methods to categorize biomolecular condensate function

Level of study	Technique	Description	Benefits	Disadvantages	References or examples
Organismal	Functional complementation of a null mutant with a noncondensing protein in the native system	Expressing a mutant protein that cannot form condensates driven by the native promoter in the null mutant and seeing whether it cannot rescue the null phenotype.	Provides strong information if the condensate is critical for function.	Need structural information of protein and how to disrupt condensate formation, is slow to generate, need to phenotype.	Emenecker et al. (2021)
	Transient expression	Expressing the protein transiently in a plant system (e.g. protoplasts, tobacco).	Is quick, and can test which protein domains are essential for condensate formation.	Biologically relevant proteins, stresses, or other unknown parameters that may be required in the native system cannot be replicated.	Emenecker et al. (2021)
	Heterologous expression and in vitro analysis	Expressing the protein in a non-native system (e.g. yeast, human cells) and testing the protein/condensate function in vitro.	Can generate functional information, can be quicker than generating a stable plant line.	Same issues as the transient expression above. Need to optimize expression, and need to know what function to test.	Emenecker et al. (2021), Alberti et al. (2019)
Cellular and subcellular	Fluorescence recovery after photobleaching (FRAP)	Bleaching part of a fluorescently labeled condensate and measuring how quickly fluorescence diffuses back into the bleached area. Determines if the condensate is formed by LLPS.	Measure fluidity of a condensate, can compare viscosity of condensates relative to each other.	Condensate properties can differ between expression systems and expression levels.	Emenecker et al. (2021), Ganser et al. (2023), Alberti et al. (2019)
	1,6-Hexanediol	Treating cells with 1,6-hexanediol to see if the biomolecular condensate dissolves.	Can be informative for quickly disrupting LLPS and inferring formation through LLPS.	Can trigger granule formation with prolonged incubation (> a few minutes).	Alberti et al. (2019)
	Stochastic optical reconstruction microscopy (STORM)	Super resolution single molecule fluorescence.	Can be used to test for sub cellular localization of condensates.	Time consuming.	Feng et al. (2019)
	Transmission electron microscopy (TEM)	High resolution imaging of biomolecular condensates in their native system.	Can see nanoscale structure of biomolecular condensates in the native system.	Need to fix samples, which may result in non-native structures or localizaiton.	Hamada et al. (2018) and Bounedjah et al. (2014)
Molecular	Total internal reflection fluorescence (TIRF) microscopy	High resolution and shallow depth fluorescence microscopy that allows single molecule analysis. Used for determining changes in dwell time.	Can measure single molecule interactions.	Need purified proteins.	Feng et al. (2019)
	Cryogenic electron microscopy (Cryo-EM) and tomography (Cryo-ET)	Electron microscopy and tomography techniques on frozen samples, useful for viewing the native structure of proteins and condensates.	Can get high resolution structures of biomolecular condensates in their native states.	Need purified proteins for Cryo-EM. Cryo-ET not well worked out in plants.	Tollervey et al. (2023)
Biophysical	Time domain NMR (TD-NMR)	Nuclear Magnetic Resonance technique that measures water coordination within a tissue.	Can accurately measure water coordination and changes in the state of water in biological samples.	Need a large amount of biological samples.	Hesgrove et al. (2021)

### Open questions

- What developmental processes in plants require biomolecular condensate formation?
- Do environmental conditions change development directly through changes in biomolecular condensate formation and condensate properties?
- What are the physical properties of biomolecular condensates in different environments, how do they vary across a plant's life cycle, and do these changes impact function?
- Are there functions of biomolecular condensates outside of the broad mechanisms of sequestration, increasing dwell time, and modulating cellular biophysical properties?
- What protein domains are critical for condensate formation and how diverse are mechanisms of condensate formation?
- What roles do RNA and RNA processing play in biomolecular condensate formation and dissolution during development?

### How can functional mechanisms of biomolecular condensates be tested?

We discussed several well-established developmental regulators, which were recently demonstrated to undergo condensate formation. The ongoing challenge in the field is to understand if condensation is critical for the function of these proteins and pathways. The diversity of protein and condensate function makes it difficult to understand the specific molecular function of a given protein, though we suggest 2 guiding principles: (i) the function of the biomolecular condensate can be tested by categorizing it into one of the 3 general mechanisms (Fig. 1) to inform researchers to make hypothesis driven questions; and (ii) connecting condensate formation with function can be tested using mutants that can no longer form condensates. We have highlighted examples of several protein domains critical for condensate formation (see Table 1). Future work should mutate these protein domains to prevent condensate formation and use these mutants to test if condensate formation is critical for development. Successful examples of this approach include mutations to the ARF19 PB1 domain (ARF19^K962A^; Powers et al. 2019) and mutations to the FLOE1 DS and QPS domains (Dorone et al. 2021).

### How can we increase the speed of research connecting condensate formation to function in plants?

Molecular insight into biomolecular condensate functions in plants lags behind human and yeast systems due to the relatively long generation time for making stable reporter lines in plants. To facilitate a faster pace of biomolecular condensate research in plants, we propose the use of transient expression systems to: (i) understand if the protein can form a biomolecular condensate; and (ii) identify the protein domains essential for condensate formation. Additionally, transient systems can be used for generating a rough understanding of protein colocalization to specific condensate populations with conserved markers, though it should be used with caution (further discussed below).

### When are transient systems inappropriate for investigating biomolecular condensates?

One caution in biomolecular condensate research is that condensate formation can be regulated by protein concentration. Overexpression of biomolecular condensate proteins generally causes condensation that is different from those of the endogenous system (Alberti et al. 2019; Guillén-Boixet et al. 2020). For this reason, testing biomolecular condensation in the endogenous system is critical to characterize biologically relevant biochemical and biophysical properties of the condensate and accurately understand their formation and function. This was demonstrated by Xie et al. (2021) who showed DCL1, HYL1, and SERRATE proteins form biomolecular condensates in *Nicotiana benthamiana* and Arabidopsis, though FRAP recovery rates identified Arabidopsis biomolecular condensates as less liquid than *N. benthamiana* condensates (Xie et al. 2021), indicating altered condensate properties. The endogenous plant system should ultimately be used to dissect specific developmental functions mediated by the biomolecular condensates. Further, transient systems can be a powerful tool if we establish how formation and properties of current plant condensates in the endogenous system compare with their formation and properties in a transient system. If condensate properties can be established in both systems, then researchers will know which biomolecular condensates are appropriate to study in transient systems and which always require studying in the native system.

### How can we study the impact of environmental cues on biomolecular condensate formation?

Studying biomolecular condensates in plants is exciting because environmental conditions readily impact condensate formation. Unfortunately, this also poses challenges because the rapid formation or dissolution of condensates can occur during processing of samples when moved to new conditions. Live-cell imaging is one of the most common microscopy techniques to study biomolecular condensates in plants and can be used to quickly study condensates in living plants. In situations where the biomolecular condensate is extremely dynamic, environmental control of the microscope stage can be essential for studying condensates in response to environmental stimuli. Humidity and temperature controlled microscope stages are now available and should help aid in the understanding of environmental inputs triggering condensate regulation in planta.

### When can we say a biomolecular condensate is formed through LLPS?

Not all biomolecular condensates are formed by LLPS (further discussed in Table 1). FRAP is typically used to test for LLPS due to the rapid diffusion of fluorescent protein back into the bleached site (Alberti et al. 2019). Other assays, like 1,6-Hexanediol treatment to test if an LLPS dissolves, have been used to test for hydrophobic interactions associated with LLPS, though 1,6-hexanediol treatment has been identified as increasing LLPS formation in some conditions in vivo (Wheeler et al. 2016). In general, establishing that a condensate forms through LLPS requires additional lines of evidence, and researchers wanting to do so should review current methodologies (Alberti et al. 2019; Ganser and Myong 2020; Gao et al. 2022; Wang et al. 2022).

### How can experimental data from across eukaryotes benefit plant-specific biomolecular condensate function research?

Information generated on similar proteins in other eukaryotes can provide a useful model to test the function of plant-specific proteins, particularly in condensates like stress granules and processing bodies. Additionally, an interesting aspect of biomolecular condensate research in plants is the identification of proteins that form biomolecular condensates, which have previously been linked to organismal level phenotypes. In contrast to plant research, biomolecular condensate research in yeast and human cell lines typically starts and remains in single cell systems, making it difficult to understand the role of biomolecular condensate regulation at the organismal level. This is an excellent opportunity for the plant biomolecular condensate field because we can study proteins similar to those implicated in human diseases and disorders, which are functionally conserved with plant proteins (e.g. G3BP; Reuper et al. 2021), and connect this regulation back to the fundamental biological processes at the organismal level.

## Conclusions

Plant development is regulated by mechanisms involving biomolecular condensates at various stages of the life cycle (Fig. 2). Overall, biomolecular condensates function to sequester (FRI, ARF19, TMF) or increase local concentration (FCA, CRY2) of components that result in changes to development, with input from environmental signals. Further research into biomolecular condensate interaction with cytoplasmic biophysical properties (FLOE1, LEA) will also enhance our understanding of the biophysical mechanisms important for condensate function. Additionally, the diverse roles of condensates in plant development pose interesting future research directions to further understand the diverse strategies plants use to tailor development for changing conditions. Altogether, plants pose an exquisite system to study the regulation of development by biomolecular condensates that stand to enhance our understanding of the basics of their regulation and mechanism at the molecular, cellular, and organismal levels.

## References

[koad062-B1] Alberti S , Gladfelter A, Mittag T. Considerations and challenges in studying liquid-liquid phase separation and biomolecular condensates. Cell. 2019:176(3):419–434. 10.1016/j.cell.2018.12.035 30682370 PMC6445271

[koad062-B2] Allen KD , Sussex IM. Falsiflora and anantha control early stages of floral meristem development in tomato (Lycopersicon esculentum mill. Planta. 1996:200(2):254–264. 10.1007/BF00208316

[koad062-B3] Banani SF , Lee HO, Hyman AA, Rosen MK. Biomolecular condensates: organizers of cellular biochemistry. Nat Rev Mol Cell Biol. 2017:18(5):285–298. 10.1038/nrm.2017.7 28225081 PMC7434221

[koad062-B4] Battaglia M , Olvera-Carrillo Y, Garciarrubio A, Campos F, Covarrubias AA. The enigmatic LEA proteins and other hydrophilins. Plant Physiol. 2008:148(1):6–24. 10.1104/pp.108.120725 18772351 PMC2528095

[koad062-B5] Belott C , Janis B, Menze MA. Liquid-liquid phase separation promotes animal desiccation tolerance. Proc Natl Acad Sci USA. 2020:117(44):27676–27684. 10.1073/pnas.2014463117 33077592 PMC7959570

[koad062-B6] Berry S , Rosa S, Howard M, Bühler M, Dean C. Disruption of an RNA-binding hinge region abolishes LHP1-mediated epigenetic repression. Genes Dev. 2017:31(21):2115–2120. 10.1101/gad.305227.117 29212661 PMC5749160

[koad062-B7] Bienz M . Head-to-tail polymerization in the assembly of biomolecular condensates. Cell. 2020:182(4):799–811. 10.1016/j.cell.2020.07.037 32822572

[koad062-B8] Bounedjah O , Desforges B, Wu T-D, Pioche-Durieu C, Marco S, Hamon L, Curmi PA, Guerquin-Kern J-L, Piétrement O, Pastré D. Free mRNA in excess upon polysome dissociation is a scaffold for protein multimerization to form stress granules. Nucleic Acids Res. 2014:42(13):8678–8691. 10.1093/nar/gku582 25013173 PMC4117795

[koad062-B9] Carrera-Castaño G , Calleja-Cabrera J, Pernas M, Gómez L, Oñate-Sánchez L. An updated overview on the regulation of seed germination. Plants. 2020:9(6):703. 10.3390/plants9060703 32492790 PMC7356954

[koad062-B10] Case LB , Zhang X, Ditlev JA, Rosen MK. Stoichiometry controls activity of phase-separated clusters of actin signaling proteins. Science. 2019:363(6431):1093–1097. 10.1126/science.aau6313 30846599 PMC6784323

[koad062-B11] Chakrabortee S , Tripathi R, Watson M, Schierle GSK, Kurniawan DP, Kaminski CF, Wise MJ, Tunnacliffe A. Intrinsically disordered proteins as molecular shields. Mol Biosyst. 2012:8(1):210–219. 10.1039/C1MB05263B 21909508 PMC5365143

[koad062-B12] Chen G-H , Liu M-J, Xiong Y, Sheen J, Wu S-H. TOR And RPS6 transmit light signals to enhance protein translation in deetiolating *Arabidopsis* seedlings. Proc Natl Acad Sci USA. 2018:115(50):12823–12828. 10.1073/pnas.1809526115 30482859 PMC6294885

[koad062-B13] Chen D , Lyu M, Kou X, Li J, Yang Z, Gao L, Li Y, Fan L-M, Shi H, Zhong S. Integration of light and temperature sensing by liquid-liquid phase separation of phytochrome B. Mol Cell. 2022:82(16):3015–3029.e6. 10.1016/j.molcel.2022.05.026 35728588

[koad062-B14] Chen M , Schwab R, Chory J. Characterization of the requirements for localization of phytochrome B to nuclear bodies. Proc Natl Acad Sci USA. 2003:100(24):14493–14498. 10.1073/pnas.1935989100 14612575 PMC283619

[koad062-B15] Dang NX , Popova AV, Hundertmark M, Hincha DK. Functional characterization of selected LEA proteins from *Arabidopsis thaliana* in yeast and in vitro. Planta. 2014:240(2):325–336. 10.1007/s00425-014-2089-z 24841476

[koad062-B16] Delarue M , Brittingham GP, Pfeffer S, Surovtsev IV, Pinglay S, Kennedy KJ, Schaffer M, Gutierrez JI, Sang D, Poterewicz G, et al mTORC1 controls phase separation and the biophysical properties of the cytoplasm by tuning crowding. Cell. 2018:174(2):338–349.e20. 10.1016/j.cell.2018.05.042 29937223 PMC10080728

[koad062-B17] Deng W , Ying H, Helliwell CA, Taylor JM, Peacock WJ, Dennis ES. FLOWERING LOCUS C (FLC) regulates development pathways throughout the life cycle of *Arabidopsis*. Proc Natl Acad Sci USA. 2011:108(16):6680–6685. 10.1073/pnas.1103175108 21464308 PMC3081018

[koad062-B18] Derkacheva M , Steinbach Y, Wildhaber T, Mozgová I, Mahrez W, Nanni P, Bischof S, Gruissem W, Hennig L. *Arabidopsis* MSI1 connects LHP1 to PRC2 complexes. EMBO J. 2013:32(14):2073–2085. 10.1038/emboj.2013.145 23778966 PMC3715863

[koad062-B19] Dorone Y , Boeynaems S, Flores E, Jin B, Hateley S, Bossi F, Lazarus E, Pennington JG, Michiels E, De Decker M, et al A prion-like protein regulator of seed germination undergoes hydration-dependent phase separation. Cell. 2021:184(16):4284–4298.e27. 10.1016/j.cell.2021.06.009 34233164 PMC8513799

[koad062-B20] Emenecker RJ , Holehouse AS, Strader LC. Biological phase separation and biomolecular condensates in plants. Annu Rev Plant Biol. 2021:72(1):17–46. 10.1146/annurev-arplant-081720-015238 33684296 PMC8221409

[koad062-B21] Fang X , Wang L, Ishikawa R, Li Y, Fiedler M, Liu F, Calder G, Rowan B, Weigel D, Li P, et al *Arabidopsis* FLL2 promotes liquid-liquid phase separation of polyadenylation complexes. Nature. 2019:569(7755):265–269. 10.1038/s41586-019-1165-8 31043738 PMC6625965

[koad062-B22] Fang X , Wu Z, Raitskin O, Webb K, Voigt P, Lu T, Howard M, Dean C. The 3′ processing of antisense RNAs physically links to chromatin-based transcriptional control. Proc Natl Acad Sci USA. 2020:117(26):15316–15321. 10.1073/pnas.2007268117 32541063 PMC7334503

[koad062-B23] Feng Z , Chen X, Wu X, Zhang M. Formation of biological condensates via phase separation: characteristics, analytical methods, and physiological implications. J Biol Chem. 2019:294(40):14823–14835. 10.1074/jbc.REV119.007895 31444270 PMC6779427

[koad062-B24] Fiedler M , Franco-Echevarría E, Schulten A, Nielsen M, Rutherford TJ, Yeates A, Ahsan B, Dean C, Bienz M. Head-to-tail polymerization by VEL proteins underpins cold-induced polycomb silencing in flowering control. Cell Rep. 2022:41(6):111607. 10.1016/j.celrep.2022.111607 36351412 PMC7614096

[koad062-B25] Galvão VC , Fankhauser C. Sensing the light environment in plants: photoreceptors and early signaling steps. Curr Opin Neurobiol. 2015:34:46–53. 10.1016/j.conb.2015.01.013 25638281

[koad062-B26] Ganser LR , Ge Y, Myong S. Single-molecule fluorescence methods to study protein-RNA interactions underlying biomolecular condensates. Methods Mol. Biol. 2023:2563:149–160. 10.1007/978-1-0716-2663-4_7 36227472

[koad062-B27] Ganser LR , Myong S. Methods to study phase-separated condensates and the underlying molecular interactions. Trends Biochem Sci. 2020:45(11):1004–1005. 10.1016/j.tibs.2020.05.011 32561165 PMC7697221

[koad062-B28] Gao Y , Li X, Li P, Lin Y. A brief guideline for studies of phase-separated biomolecular condensates. Nat Chem Biol. 2022:18(12):1307–1318. 10.1038/s41589-022-01204-2 36400991

[koad062-B29] Ginsawaeng O , Heise C, Sangwan R, Karcher D, Hernández-Sánchez IE, Sampathkumar A, Zuther E. Subcellular localization of seed-expressed LEA_4 proteins reveals liquid-liquid phase separation for LEA9 and for LEA48 homo- and LEA42-LEA48 heterodimers. Biomolecules. 2021:11(12):1770. 10.3390/biom11121770 34944414 PMC8698616

[koad062-B30] Goyal K , Walton LJ, Tunnacliffe A. LEA proteins prevent protein aggregation due to water stress. Biochem J. 2005:388(1):151–157. 10.1042/BJ20041931 15631617 PMC1186703

[koad062-B31] Greb T , Mylne JS, Crevillen P, Geraldo N, An H, Gendall AR, Dean C. The PHD finger protein VRN5 functions in the epigenetic silencing of *Arabidopsis* FLC. Curr Biol. 2007:17(1):73–78. 10.1016/j.cub.2006.11.052 17174094

[koad062-B32] Grigg SP , Canales C, Hay A, Tsiantis M. SERRATE coordinates shoot meristem function and leaf axial patterning in *Arabidopsis*. Nature. 2005:437(7061):1022–1026. 10.1038/nature04052 16222298

[koad062-B33] Guillén-Boixet J , Kopach A, Holehouse AS, Wittmann S, Jahnel M, Schlüßler R, Kim K, Trussina IREA, Wang J, Mateju D, et al RNA-induced conformational switching and clustering of G3BP drive stress granule assembly by condensation. Cell. 2020:181(2):346–361.e17. 10.1016/j.cell.2020.03.049 32302572 PMC7181197

[koad062-B34] Hamada T , Yako M, Minegishi M, Sato M, Kamei Y, Yanagawa Y, Toyooka K, Watanabe Y, Hara-Nishimura I. Stress granule formation is induced by a threshold temperature rather than a temperature difference in *Arabidopsis*. J Cell Sci. 2018:131(16):jcs216051. 10.1242/jcs.216051 30030372

[koad062-B35] Helliwell CA , Wood CC, Robertson M, James Peacock W, Dennis ES. The *Arabidopsis* FLC protein interacts directly in vivo with SOC1 and FT chromatin and is part of a high-molecular-weight protein complex. Plant J. 2006:46(2):183–192. 10.1111/j.1365-313X.2006.02686.x 16623882

[koad062-B36] Herrero E , Kolmos E, Bujdoso N, Yuan Y, Wang M, Berns MC, Uhlworm H, Coupland G, Saini R, Jaskolski M, et al EARLY FLOWERING4 recruitment of EARLY FLOWERING3 in the nucleus sustains the *Arabidopsis* circadian clock. Plant Cell. 2012:24(2):428–443. 10.1105/tpc.111.093807 22327739 PMC3315225

[koad062-B37] Hesgrove C , Boothby TC. The biology of tardigrade disordered proteins in extreme stress tolerance. Cell Commun Signal. 2020:18(1):178. 10.1186/s12964-020-00670-2 33148259 PMC7640644

[koad062-B38] Hesgrove CS , Nguyen KH, Biswas S, Childs CA, Shraddha KC, Medina BX, Alvarado V, Yu F, Sukenik S, Malferrari M, et al Tardigrade CAHS proteins act as molecular Swiss Army knives to mediate desiccation tolerance through multiple mechanisms. bioRxiv: 2021.08.16.456555. 10.1101/2021.08.16.456555 , 15 November 2021, preprint: not peer reviewed.

[koad062-B39] Hofmann S , Kedersha N, Anderson P, Ivanov P. Molecular mechanisms of stress granule assembly and disassembly. Biochim Biophys Acta Mol Cell Res. 2021:1868(1):118876. 10.1016/j.bbamcr.2020.118876 33007331 PMC7769147

[koad062-B40] Huang X , Chen S, Li W, Tang L, Zhang Y, Yang N, Zou Y, Zhai X, Xiao N, Liu W, et al ROS regulated reversible protein phase separation synchronizes plant flowering. Nat Chem Biol. 2021:17(5):549–557. 10.1038/s41589-021-00739-0 33633378

[koad062-B41] Hundertmark M , Hincha DK. LEA (late embryogenesis abundant) proteins and their encoding genes in *Arabidopsis thaliana*. BMC Genomics. 2008:9(1):118. 10.1186/1471-2164-9-118 18318901 PMC2292704

[koad062-B42] Jang G-J , Jang J-C, Wu S-H. Dynamics and functions of stress granules and processing bodies in plants. Plants. 2020:9(9):1122. 10.3390/plants9091122 32872650 PMC7570210

[koad062-B43] Jang G-J , Yang J-Y, Hsieh H-L, Wu S-H. Processing bodies control the selective translation for optimal development of *Arabidopsis* young seedlings. Proc Natl Acad Sci USA. 2019:116(13):6451–6456. 10.1073/pnas.1900084116 30850529 PMC6442596

[koad062-B44] Jing H , Korasick DA, Emenecker RJ, Morffy N, Wilkinson EG, Powers SK, Strader LC. Regulation of AUXIN RESPONSE FACTOR condensation and nucleo-cytoplasmic partitioning. Nat Commun. 2022:13(1):4015. 10.1038/s41467-022-31628-2 35817767 PMC9273615

[koad062-B45] Jung J-H , Barbosa AD, Hutin S, Kumita JR, Gao M, Derwort D, Silva CS, Lai X, Pierre E, Geng F, et al A prion-like domain in ELF3 functions as a thermosensor in *Arabidopsis*. Nature. 2020:585(7824):256–260. 10.1038/s41586-020-2644-7 32848244

[koad062-B46] Kaiserli E , Páldi K, O’Donnell L, Batalov O, Pedmale UV, Nusinow DA, Kay SA, Chory J. Integration of light and photoperiodic signaling in transcriptional nuclear foci. Dev Cell. 2015:35(3):311–321. 10.1016/j.devcel.2015.10.008 26555051 PMC4654455

[koad062-B47] Kim G-T , Shoda K, Tsuge T, Cho K-H, Uchimiya H, Yokoyama R, Nishitani K, Tsukaya H. The ANGUSTIFOLIA gene of *Arabidopsis*, a plant CtBP gene, regulates leaf-cell expansion, the arrangement of cortical microtubules in leaf cells and expression of a gene involved in cell-wall formation. EMBO J. 2002:21(6):1267–1279. 10.1093/emboj/21.6.1267 11889033 PMC125914

[koad062-B48] Kim EY , Wang L, Lei Z, Li H, Fan W, Cho J. Ribosome stalling and SGS3 phase separation prime the epigenetic silencing of transposons. Nat Plants. 2021:7(3):303–309. 10.1038/s41477-021-00867-4 33649597

[koad062-B49] Kircher S , Kozma-Bognar L, Kim L, Adam E, Harter K, Schäfer E, Nagy F. Light quality–dependent nuclear import of the plant photoreceptors phytochrome A and B. Plant Cell. 1999:11(8):1445–1456. 10.1105/tpc.11.8.1445 10449579 PMC144301

[koad062-B50] Kohchi T , Mukougawa K, Frankenberg N, Masuda M, Yokota A, Lagarias JC. The *Arabidopsis HY2* gene encodes phytochromobilin synthase, a ferredoxin-dependent biliverdin reductase. Plant Cell. 2001:13(2):425–436. 10.1105/tpc.13.2.425 11226195 PMC102252

[koad062-B51] Koornneef M , Meinke D. The development of *Arabidopsis* as a model plant. Plant J. 2010:61(6):909–921. 10.1111/j.1365-313X.2009.04086.x 20409266

[koad062-B52] Lau OS , Deng XW. The photomorphogenic repressors COP1 and DET1: 20 years later. Trends Plant Sci. 2012:17(10):584–593. 10.1016/j.tplants.2012.05.004 22705257

[koad062-B53] Levy YY , Mesnage S, Mylne JS, Gendall AR, Dean C. Multiple roles of *Arabidopsis VRN1* in vernalization and flowering time control. Science. 2002:297(5579):243–246. 10.1126/science.1072147 12114624

[koad062-B54] Lippman ZB , Cohen O, Alvarez JP, Abu-Abied M, Pekker I, Paran I, Eshed Y, Zamir D. The making of a compound inflorescence in tomato and related nightshades. PLoS Biol. 2008:6(11):e288. 10.1371/journal.pbio.0060288 19018664 PMC2586368

[koad062-B55] Liu F , Marquardt S, Lister C, Swiezewski S, Dean C. Targeted 3′ processing of antisense transcripts triggers *Arabidopsis* FLC chromatin silencing. Science. 2010:327(5961):94–97. 10.1126/science.1180278 19965720

[koad062-B56] Lobbes D , Rallapalli G, Schmidt DD, Martin C, Clarke J. SERRATE: a new player on the plant microRNA scene. EMBO Rep. 2006:7(10):1052–1058. 10.1038/sj.embor.7400806 16977334 PMC1618363

[koad062-B57] Lorković ZJ . Role of plant RNA-binding proteins in development, stress response and genome organization. Trends Plant Sci. 2009:14(4):229–236. 10.1016/j.tplants.2009.01.007 19285908

[koad062-B58] Luo Y , Na Z, Slavoff SA. P-bodies: composition, properties, and functions. Biochemistry. 2018:57(17):2424–2431. 10.1021/acs.biochem.7b01162 29381060 PMC6296482

[koad062-B59] Lyon AS , Peeples WB, Rosen MK. A framework for understanding the functions of biomolecular condensates across scales. Nat Rev Mol Cell Biol. 2020:22(3):215–235. 10.1038/s41580-020-00303-z 33169001 PMC8574987

[koad062-B60] Mackenzie JM Jr , Coleman RA, Briggs WR, Pratt LH. Reversible redistribution of phytochrome within the cell upon conversion to its physiologically active form. Proc Natl Acad Sci USA. 1975:72(3):799–803. 10.1073/pnas.72.3.799 1093170 PMC432407

[koad062-B61] Más P , Devlin PF, Panda S, Kay SA. Functional interaction of phytochrome B and cryptochrome 2. Nature. 2000:408(6809):207–211. 10.1038/35041583 11089975

[koad062-B62] Merchante C , Stepanova AN, Alonso JM. Translation regulation in plants: an interesting past, an exciting present and a promising future. Plant J. 2017:90(4):628–653. 10.1111/tpj.13520 28244193

[koad062-B63] Millar AJ . Input signals to the plant circadian clock. J Exp Bot. 2004:55(395):277–283. 10.1093/jxb/erh034 14695902

[koad062-B64] Mo W , Zhang J, Zhang L, Yang Z, Yang L, Yao N, Xiao Y, Li T, Li Y, Zhang G, et al *Arabidopsis* cryptochrome 2 forms photobodies with TCP22 under blue light and regulates the circadian clock. Nat Commun. 2022:13(1):2631. 10.1038/s41467-022-30231-9 35551190 PMC9098493

[koad062-B65] Murcia G , Nieto C, Sellaro R, Prat S, Casal JJ. Hysteresis in PHYTOCHROME-INTERACTING FACTOR 4 and EARLY-FLOWERING 3 dynamics dominates warm daytime memory in *Arabidopsis*. Plant Cell. 2022:34(6):2188–2204. 10.1093/plcell/koac078 35234947 PMC9134080

[koad062-B66] Mylne JS , Barrett L, Tessadori F, Mesnage S, Johnson L, Bernatavichute YV, Jacobsen SE, Fransz P, Dean C. LHP1, the *Arabidopsis* homologue of HETEROCHROMATIN PROTEIN1, is required for epigenetic silencing of *FLC*. Proc Natl Acad Sci USA. 2006:103(13):5012–5017. 10.1073/pnas.0507427103 16549797 PMC1458786

[koad062-B67] Nonogaki H . Seed dormancy and germination-emerging mechanisms and new hypotheses. Front Plant Sci. 2014:5:233. 10.3389/fpls.2014.00233 24904627 PMC4036127

[koad062-B68] Nusinow DA , Helfer A, Hamilton EE, King JJ, Imaizumi T, Schultz TF, Farré EM, Kay SA. The ELF4–ELF3–LUX complex links the circadian clock to diurnal control of hypocotyl growth. Nature. 2011:475(7356):398–402. 10.1038/nature10182 21753751 PMC3155984

[koad062-B69] Powers SK , Holehouse AS, Korasick DA, Schreiber KH, Clark NM, Jing H, Emenecker R, Han S, Tycksen E, Hwang I, et al Nucleo-cytoplasmic partitioning of ARF proteins controls auxin responses in *Arabidopsis thaliana*. Mol Cell. 2019:76(1):177–190.e5. 10.1016/j.molcel.2019.06.044 31421981 PMC6778021

[koad062-B70] Prigge MJ , Wagner DR. The *Arabidopsis* serrate gene encodes a zinc-finger protein required for normal shoot development. Plant Cell. 2001:13(6):1263–1279. 10.1105/TPC.010095 11402159 PMC135584

[koad062-B71] Protter DSW , Parker R. Principles and properties of stress granules. Trends Cell Biol. 2016:26(9):668–679. 10.1016/j.tcb.2016.05.004 27289443 PMC4993645

[koad062-B72] Rajjou L , Duval M, Gallardo K, Catusse J, Bally J, Job C, Job D. Seed germination and vigor. Annu Rev Plant Biol. 2012:63(1):507–533. 10.1146/annurev-arplant-042811-105550 22136565

[koad062-B73] Reuper H , Götte B, Williams L, Tan TJC, McInerney GM, Panas MD, Krenz B. *Arabidopsis thaliana* G3BP ortholog rescues mammalian stress granule phenotype across kingdoms. Int J Mol Sci. 2021:22(12):6287. 10.3390/ijms22126287 34208100 PMC8230867

[koad062-B74] Roden C , Gladfelter AS. RNA contributions to the form and function of biomolecular condensates. Nat Rev Mol Cell Biol. 2020:22(3):183–195. 10.1038/s41580-020-0264-6 32632317 PMC7785677

[koad062-B75] Ronald J , Su C, Wang L, Davis SJ. Cellular localization of *Arabidopsis* EARLY FLOWERING3 is responsive to light quality. Plant Physiol. 2022:190(2):1024–1036. 10.1093/plphys/kiac072 35191492 PMC9516731

[koad062-B76] Saijo Y , Sullivan JA, Wang H, Yang J, Shen Y, Rubio V, Ma L, Hoecker U, Deng XW. The COP1-SPA1 interaction defines a critical step in phytochrome A-mediated regulation of HY5 activity. Genes Dev. 2003:17(21):2642–2647. 10.1101/gad.1122903 14597662 PMC280614

[koad062-B77] Sakamoto K , Nagatani A. Nuclear localization activity of phytochrome B. Plant J. 1996:10(5):859–868. 10.1046/j.1365-313X.1996.10050859.x 8953247

[koad062-B78] Schwenk P , Hiltbrunner A. Phytochrome A mediates the disassembly of processing bodies in far-red light. Front Plant Sci. 2022:13:828529. 10.3389/fpls.2022.828529 35283917 PMC8905148

[koad062-B79] Schwenk P , Sheerin DJ, Ponnu J, Staudt A-M, Lesch KL, Lichtenberg E, Medzihradszky KF, Hoecker U, Klement E, Viczián A, et al Uncovering a novel function of the CCR4-NOT complex in phytochrome A-mediated light signalling in plants. eLife. 2021:10:e63697. 10.7554/eLife.63697 33783355 PMC8009681

[koad062-B80] Searle I , He Y, Turck F, Vincent C, Fornara F, Kröber S, Amasino RA, Coupland G. The transcription factor FLC confers a flowering response to vernalization by repressing meristem competence and systemic signaling in *Arabidopsis*. Genes Dev. 2006:20(7):898–912. 10.1101/gad.373506 16600915 PMC1472290

[koad062-B81] Song X , Li Y, Cao X, Qi Y. MicroRNAs and their regulatory roles in plant–environment interactions. Annu Rev Plant Biol. 2019:70(1):489–525. 10.1146/annurev-arplant-050718-100334 30848930

[koad062-B82] Sun Q , Csorba T, Skourti-Stathaki K, Proudfoot NJ, Dean C. R-loop stabilization represses antisense transcription at the *Arabidopsis* FLC locus. Science. 2013:340(6132):619–621. 10.1126/science.1234848 23641115 PMC5144995

[koad062-B83] Sung S , Amasino RM. Vernalization in *Arabidopsis thaliana* is mediated by the PHD finger protein VIN3. Nature. 2004:427(6970):159–164. 10.1038/nature02195 14712276

[koad062-B84] Sung S , He Y, Eshoo TW, Tamada Y, Johnson L, Nakahigashi K, Goto K, Jacobsen SE, Amasino RM. Epigenetic maintenance of the vernalized state in *Arabidopsis thaliana* requires LIKE HETEROCHROMATIN PROTEIN 1. Nat Genet. 2006:38(6):706–710. 10.1038/ng1795 16682972

[koad062-B85] Tanaka A , Nakano T, Watanabe K, Masuda K, Honda G, Kamata S, Yasui R, Kozuka-Hata H, Watanabe C, Chinen T, et al Stress-dependent cell stiffening by tardigrade tolerance proteins that reversibly form a filamentous network and gel. PLoS Biol. 2022:20(9):e3001780. 10.1371/journal.pbio.3001780 36067153 PMC9592077

[koad062-B86] Tollervey F , Zhang X, Bose M, Sachweh J, Woodruff JB, Franzmann TM, Mahamid J. Cryo-electron tomography of reconstituted biomolecular condensates. In: Zhou H-X, Spille J-H, Banerjee PR, editors. Phase-separated biomolecular condensates: methods and protocols. New York (NY): Springer US; 2023. p. 297–324. 10.1007/978-1-0716-2663-4_15 36227480

[koad062-B87] Tunnacliffe A , Wise MJ. The continuing conundrum of the LEA proteins. Naturwissenschaften. 2007:94(10):791–812. 10.1007/s00114-007-0254-y 17479232

[koad062-B88] van Dop M , Fiedler M, Mutte S, de Keijzer J, Olijslager L, Albrecht C, Liao C-Y, Janson ME, Bienz M, Weijers D. DIX domain polymerization drives assembly of plant cell polarity complexes. Cell. 2020:180(3):427–439.e12. 10.1016/j.cell.2020.01.011 32004461 PMC7042713

[koad062-B89] Wang X , Jiang B, Gu L, Chen Y, Mora M, Zhu M, Noory E, Wang Q, Lin C. A photoregulatory mechanism of the circadian clock in *Arabidopsis*. Nat Plants. 2021a:7(10):1397–1408. 10.1038/s41477-021-01002-z 34650267

[koad062-B90] Wang Q , Lin C. Mechanisms of cryptochrome-mediated photoresponses in plants. Annu Rev Plant Biol. 2020:71(1):103–129. 10.1146/annurev-arplant-050718-100300 32169020 PMC7428154

[koad062-B91] Wang Z , Lou J, Zhang H. Essence determines phenomenon: assaying the material properties of biological condensates. J Biol Chem. 2022:298(4):101782. 10.1016/j.jbc.2022.101782 35245500 PMC8958544

[koad062-B92] Wang Z-Y , Tobin EM. Constitutive expression of the CIRCADIAN CLOCK ASSOCIATED 1 (CCA1) gene disrupts circadian rhythms and suppresses its own expression. Cell. 1998:93(7):1207–1217. 10.1016/S0092-8674(00)81464-6 9657153

[koad062-B93] Wang Y , Zhou H, Sun X, Huang Q, Li S, Liu Z, Zhang C, Lai L. Charge segregation in the intrinsically disordered region governs VRN1 and DNA liquid-like phase separation robustness. J Mol Biol. 2021b:433(22):167269. 10.1016/j.jmb.2021.167269 34571015

[koad062-B94] Wheeler JR , Matheny T, Jain S, Abrisch R, Parker R. Distinct stages in stress granule assembly and disassembly. eLife. 2016:5:e18413. 10.7554/eLife.18413 27602576 PMC5014549

[koad062-B95] Wu J-F , Tsai H-L, Joanito I, Wu Y-C, Chang C-W, Li Y-H, Wang Y, Hong JC, Chu J-W, Hsu C-P, et al LWD-TCP complex activates the morning gene CCA1 in *Arabidopsis*. Nat Commun. 2016:7(1):13181. 10.1038/ncomms13181 27734958 PMC5065627

[koad062-B96] Xie D , Chen M, Niu J, Wang L, Li Y, Fang X, Li P, Qi Y. Phase separation of SERRATE drives dicing body assembly and promotes miRNA processing in *Arabidopsis*. Nat Cell Biol. 2021:23(1):32–39. 10.1038/s41556-020-00606-5 33288888

[koad062-B97] Xu J , Chua N-H. *Arabidopsis* decapping 5 is required for mRNA decapping, P-body formation, and translational repression during postembryonic development. Plant Cell. 2009:21(10):3270–3279. 10.1105/tpc.109.070078 19855049 PMC2782270

[koad062-B98] Xu C , Fang X, Lu T, Dean C. Antagonistic cotranscriptional regulation through ARGONAUTE1 and the THO/TREX complex orchestrates *FLC* transcriptional output. Proc Natl Acad Sci USA. 2021a:118(47):e2113757118. 10.1073/pnas.2113757118 PMC8617408 34789567

[koad062-B99] Xu C , Wu Z, Duan H-C, Fang X, Jia G, Dean C. R-loop resolution promotes co-transcriptional chromatin silencing. Nat Commun. 2021b:12(1):1790. 10.1038/s41467-021-22083-6 33741984 PMC7979926

[koad062-B100] Yang H , Berry S, Olsson TSG, Hartley M, Howard M, Dean C. Distinct phases of polycomb silencing to hold epigenetic memory of cold in *Arabidopsis*. Science. 2017:357(6356):1142–1145. 10.1126/science.aan1121 28818969

[koad062-B101] Yoshida S , van der Schuren A, van Dop M, van Galen L, Saiga S, Adibi M, Möller B, Ten Hove CA, Marhavy P, Smith R, et al A SOSEKI-based coordinate system interprets global polarity cues in *Arabidopsis*. Nat Plants. 2019:5(2):160–166. 10.1038/s41477-019-0363-6 30737509 PMC6420093

[koad062-B102] Zhang Y , Fan S, Hua C, Teo ZWN, Kiang JX, Shen L, Yu H. Phase separation of HRLP regulates flowering time in *Arabidopsis*. Sci Adv. 2022:8(25):eabn5488. 10.1126/sciadv.abn5488 35731874 PMC9217094

[koad062-B103] Zhu P , Lister C, Dean C. Cold-induced *Arabidopsis* FRIGIDA nuclear condensates for *FLC* repression. Nature. 2021:599(7886):657–661. 10.1038/s41586-021-04062-5 34732891 PMC8612926

